# Comparative analysis of root transcriptomes from two contrasting drought-responsive Williams 82 and DT2008 soybean cultivars under normal and dehydration conditions

**DOI:** 10.3389/fpls.2015.00551

**Published:** 2015-08-07

**Authors:** Chien Van Ha, Yasuko Watanabe, Uyen Thi Tran, Dung Tien Le, Maho Tanaka, Kien Huu Nguyen, Motoaki Seki, Dong Van Nguyen, Lam-Son Phan Tran

**Affiliations:** ^1^Signaling Pathway Research Unit, RIKEN Center for Sustainable Resource ScienceYokohama, Japan; ^2^National Key Laboratory for Plant Cell Technology, Agricultural Genetics Institute, Vietnamese Academy of Agricultural ScienceHanoi, Vietnam; ^3^Plant Genomic Network Research Team, RIKEN Center for Sustainable Resource ScienceYokohama, Japan; ^4^CREST, Japan Science and Technology AgencyKawaguchi, Japan

**Keywords:** soybean, dehydration, root microarray, differential expression, differential drought tolerability

## Abstract

The economically important DT2008 and the model Williams 82 (W82) soybean cultivars were reported to have differential drought-tolerant degree to dehydration and drought, which was associated with root trait. Here, we used 66K Affymetrix Soybean Array GeneChip to compare the root transcriptomes of DT2008 and W82 seedlings under normal, as well as mild (2 h treatment) and severe (10 h treatment) dehydration conditions. Out of the 38172 soybean genes annotated with high confidence, 822 (2.15%) and 632 (1.66%) genes showed altered expression by dehydration in W82 and DT2008 roots, respectively, suggesting that a larger machinery is required to be activated in the drought-sensitive W82 cultivar to cope with the stress. We also observed that long-term dehydration period induced expression change of more genes in soybean roots than the short-term one, independently of the genotypes. Furthermore, our data suggest that the higher drought tolerability of DT2008 might be attributed to the higher number of genes induced in DT2008 roots than in W82 roots by early dehydration, and to the expression changes of more genes triggered by short-term dehydration than those by prolonged dehydration in DT2008 roots vs. W82 roots. Differentially expressed genes (DEGs) that could be predicted to have a known function were further analyzed to gain a basic understanding on how soybean plants respond to dehydration for their survival. The higher drought tolerability of DT2008 vs. W82 might be attributed to differential expression in genes encoding osmoprotectant biosynthesis-, detoxification- or cell wall-related proteins, kinases, transcription factors and phosphatase 2C proteins. This research allowed us to identify genetic components that contribute to the improved drought tolerance of DT2008, as well as provide a useful genetic resource for in-depth functional analyses that ultimately leads to development of soybean cultivars with improved tolerance to drought.

## Introduction

Soybean (*Glycine max* L.) has been regarded as one of the major legume crops worldwide with multibillion dollars in value. Its seed product provides a substantial source of vegetable protein and oil, micronutrients and minerals for animal feed and human consumption (Tran and Nguyen, 2009; Choudhary and Tran, 2011). In the last several years, soybean has also shown its increasing importance in industry by supplying materials for production of biodiesel, plastics, lubricants, and hydraulic fluids (Hsien, 2015). Unfortunately, like many other crops, soybean's growth and development, and thus its productivity, are severely affected by various environmental stresses, especially drought that can cause yield loss by approximately 11–50% in various countries, including Vietnam (Vinh et al., 2010; Sadeghipour and Abbasi, 2012; Ferreira Neto et al., 2013; Ku et al., 2013). Thus, in recent years, scientific community has paid a great attention to research toward understanding of mechanisms underlying soybean responses to drought, ultimately leading to development of improved drought-tolerant soybean cultivars (Tran and Mochida, 2010; Thao and Tran, 2012; Hossain et al., 2013; Deshmukh et al., 2014).

In general, to cope with drought, a number of adaptive mechanisms are activated in plants, including soybean, through various signal transduction pathways which lead to the activation of various molecular, biochemical, and physiological responses (Hadiarto and Tran, 2011; Ha et al., 2012; Hossain et al., 2013; Deshmukh et al., 2014; Karan and Subudhi, 2014; Khan et al., 2014). Studies of the mechanisms regulating these adaptive responses, as well as identification of genes involved in these mechanisms have become a great interest of the research community. Recent advances in omics technologies, especially transcriptomics, have enabled us to identify genes, gene families and pathways associated with plant responses to stresses in a systematic manner (Ma et al., 2012; Jogaiah et al., 2013; Deshmukh et al., 2014). Taking advantage of the available soybean genomic sequences and recent progress in microarray technologies (Schmutz et al., 2010; Mochida and Shinozaki, 2011), the 66K Affymetrix soybean array platform has been designed by a US consortium, which allows us to study the expression of all the putatively annotated genes in soybean at different developmental stages, under normal, abiotic, and biotic stress conditions in a relatively reliable manner (Valdes-Lopez et al., 2011; Le et al., 2012b; Wan et al., 2015).

Root development and plasticity have been identified as a key trait in plant adaptation to drought as they determine plant access to soil water. For instance, longer primary root and/or larger xylem diameters in deep roots and/or larger lateral root system are desirable root traits which help plants adapt better to drought by acquiring water from lower soil layers or foraging subsoil surface moisture (Manavalan et al., 2009; Comas et al., 2013). Thus, identification of quantitative trait loci and genes involved in determination of root traits has been regarded as an important task of research community that has interest in elucidation of molecular mechanisms regulating plant responses to drought (Manavalan et al., 2009; Comas et al., 2013; Thao et al., 2013; Satbhai et al., 2015).

In this report, we used the 66K Affymetrix soybean GeneChip to study (i) the transcriptome-wide changes in soybean dehydrated roots vs. non-dehydrated roots and (ii) analyze the genome-wide differential gene expression in the root tissues of Williams 82 (W82) and DT2008, which have differential dehydration/drought-responsive phenotype (Ha et al., 2013), under normal and dehydration conditions. W82 is a model cultivar whose genome was sequenced several years ago (Schmutz et al., 2010), while DT2008 is an economically important cultivar grown in many regions of Vietnam (Vinh et al., 2010). DT2008 was reported to display stronger tolerance to drought than W82 in a comparative analysis, which might be associated with a better root trait (Ha et al., 2013). The results of this study will enable us to identify dehydration-responsive genes in soybean roots and understand the genetic network underlying the differential drought tolerability of W82 and DT2008, as well as provide us with a list of promising candidate genes that hold potential application in development of improved drought-tolerant transgenic soybean varieties through genetic engineering.

## Results

### Microarray analysis of W82 and DT2008 root transcriptomes under normal and dehydration conditions

**Figure 1:**
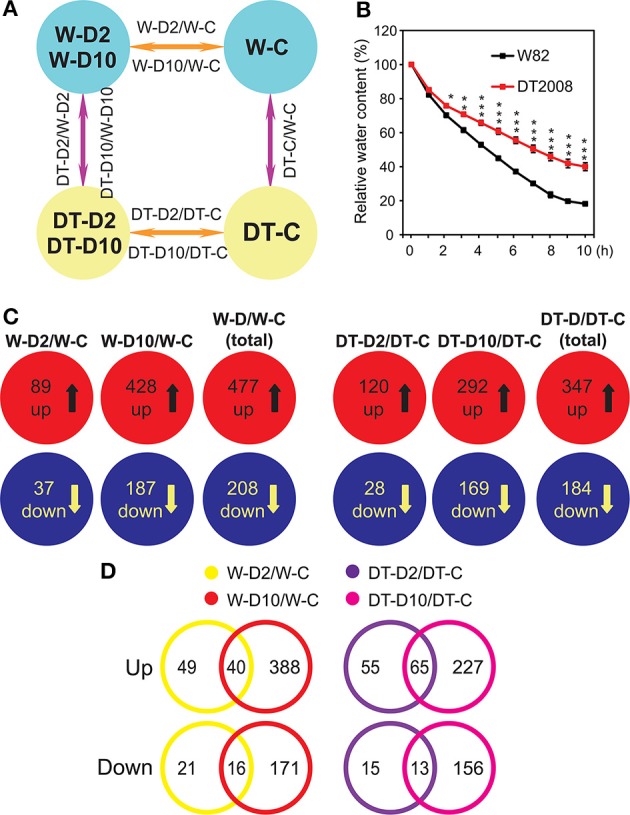
**Experimental design and summary of the results of the microarray analysis**. **(A)** Diagrams showing experimental design and comparisons. **(B)** Relative water content of W82 and DT2008 plants exposed to a dehydration treatment. Data represent the mean and SE (*n* = 5). Asterisks indicate significant differences as determined by a Student's *t*-test (^*^P < 0.05; ^**^P < 0.01 and ^***^P < 0.001). **(C)** Upregulated and downregulated genes identified in each comparison from 34097 genes that were assigned with a putative function. Data were obtained from the results of three independent microarray experiments of three biological repeats. **(D)** Effect of stress intensity on gene expression in roots of W82 and DT2008 as indicated by Venn analysis of differentially expressed gene sets identified in **(C)**. W-D2/W-C, W82-dehydrated-2 h vs. W82-well-watered control-0 h; W-D10/W-C, W82-dehydrated-10 h vs. W82-well-watered control-0 h; W-D/W-C represents W-D2/W-C and/or W-D10/W-C (W82-dehydrated-2 h and/or 10 h vs. W82-well-watered control-0 h); DT-D2/DT-C, DT2008-dehydrated-2 h vs. DT2008-well-watered control-0 h; DT-D10/DT-C, DT2008-dehydrated-10 h vs. DT2008-well-watered control-0 h; DT-D/DT-C represents DT-D2/DT-C and/or DT-D10/DT-C (DT2008-dehydrated-2 h and/or 10 h vs. DT2008-well-watered control-0 h).

In our experimental design, root transcriptomes of drought-sensitive W82 and drought-tolerant DT2008 were compared at 0 (unstressed), 2 (early stress), and 10 h (late stress) of dehydration (Figure 1A) by microarray analysis using the 66 K soybean GeneChip (Supplementary Table S1). The relative water content (RWC) of dehydrated plants was measured during dehydration treatment, and the values were 70.2 and 75.8% for W82 and DT2008, respectively, at 2 h, whereas the respective values at 10 h of dehydration were 18.1 and 40% (Figure 1B), indicating the mild and severe stress intensities. This experimental design thus allowed us to identify (i) dehydration-responsive genes in each cultivar in a time-course manner (W-D2/W-C and W-D10/W-C; DT-D2/DT-C and DT-D10/DT-C), as well as (ii) genes involved in regulatory network that regulates differential root trait (DT-C/W-C, DT-D2/W-D2, and DT-D10/W-D10), thereby potentially contributing to higher drought tolerance of DT2008 relative to W82.

Recently, the soybean genome sequence and its annotation have been substantially improved in the newest version Glyma v2.0 [Glyma.Wm82.a2.v1 (genome assembly 2 annotation version 1)] released by Phytozome 10.1 (http://phytozome.jgi.doe.gov/pz/portal.html). Using this latest Glyma v2.0 annotation, the 66K soybean GeneChip allowed us to study the expression of 38172 genes with high confidence. These genes were subjected to a search for differentially expressed genes (DEGs) using the criterion of two-fold expression change (*q* < 0.05) (Supplementary Table S2). We found that 105 and 526 genes were upregulated and 47 and 215 were downregulated in W82 roots treated with dehydration for 2 and 10 h, respectively (Supplementary Figure S1A, comparisons W-D2/W-C and W-D10/W-C; Supplementary Tables S3A–D), whereas 131 and 355 genes were upregulated and 34 and 199 were downregulated in 2 and 10 h-dehydrated DT2008 roots vs. control, respectively (Supplementary Figure S1A, comparisons DT-D2/DT-C and DT-D10/DT-C; Supplementary Tables S4A–D). A Venn analysis indicated that 50 genes were upregulated in both 2 h- and 10 h-dehydrated W82 roots, whereas 55 were upregulated by 2 h dehydration and 476 genes by 10 h dehydration only (Supplementary Figures S1A,B; Supplementary Table S3E), making a total of 581 unique genes upregulated by at least one dehydration treatment (Supplementary Figure S1A, W-D/W-C). Similarly, we found an overlap of 21 downregulated genes in roots of W82 treated with dehydration for 2 and 10 h, and a list of 241 unique genes downregulated in dehydrated W82 roots under these two treatment conditions (Supplementary Figures S1A,B; Supplementary Table S3F). As for the drought-tolerant DT2008, we noted from the Venn diagrams that 71 upregulated and 16 downregulated genes were overlapped between DT-D2/DT-C and DT-D10/DT-C comparisons, while totally 415 and 217 unique genes were upregulated and downregulated, respectively, in 2 and/or 10 h-dehydrated DT2008 roots (Supplementary Figures S1A,B, comparison DT-D/DT-C; Supplementary Tables S4E,F).

### Identification of dehydration-responsive genes with putative function in W82 and DT2008 roots

**Figure 2:**
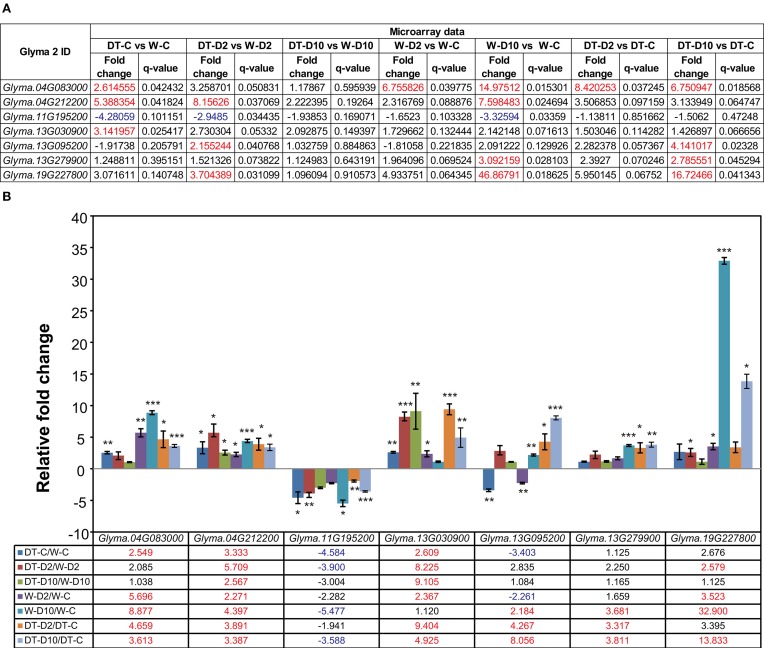
**Validation of microarray data by real-time quantitative PCR (RT-qPCR)**. Seven genes were selected for RT-qPCR verification of the microarray data. **(A)** Fold changes were obtained from microarray analysis. Red- and blue-color letters indicate absolute fold-changes ≥2 with *q* < 0.05. **(B)** Fold changes obtained by RT-qPCR of three independent biological replicates. The *Fbox* was used as reference gene. Data represent the mean plus SE (*n* = 3). Asterisks indicate significant differences as determined by a Student's *t*-test (^*^P < 0.05; ^**^P < 0.01 and ^***^P < 0.001). Red- and blue-color letters indicate absolute fold-changes ≥2 with *P* < 0.05. W-D2/W-C, W82-dehydrated-2 h vs. W82-well-watered control-0 h; W-D10/W-C, W82-dehydrated-10 h vs. W82-well-watered control-0 h; DT-D2/DT-C, DT2008-dehydrated-2 h vs. DT2008-well-watered control-0 h; DT-D10/DT-C, DT2008-dehydrated-10 h vs. DT2008-well-watered control-0 h.

Next, to identify genes modulated by dehydration in roots of W82 and/or DT2008, which have a predicted function for subsequent comparative analyses, we removed the genes with “no original description,” which are a total of 4075 genes, and examined only 34097 genes that could be assigned with a putative function (Supplementary Table S5). This approach allowed us to link the expression change by stress treatment with gene function, thereby enabling us to explain the differential root responses of W82 and DT2008 to drought. We noted 89 and 428 upregulated genes and 37 and 187 downregulated genes in W-D2/W-C and W-D10/W-C comparisons, respectively (Figure 1C; Supplementary Tables S6A–D). At the same time, we were able to detect 120 and 292 upregulated genes and 28 and 169 downregulated genes in DT-D2/DT-C and DT-D10/DT-C comparisons, respectively (Figure 1C; Supplementary Tables S7A–D). As shown by Venn analysis, 40 upregulated and 16 downregulated genes were overlapped between W-D2/W-C and W-D10/W-C comparisons, while a total of 477 and 208 unique genes were found to be upregulated and downregulated, respectively, in dehydrated W82 roots (Figure 1D; Supplementary Tables S6E,F). In case of DT2008, we detected 65 and 13 overlapped genes in the upregulated and downregulated gene sets obtained from DT-D2/DT-C and DT-D10/DT-C comparisons. Removing the overlapped genes made the lists of unique genes upregulated (347) or downregulated (184) by at least one dehydration treatment in DT2008 roots vs. control for further analyses (Figures 1C,D; Supplementary Tables S7E,F). Several genes showing various degrees of induction and repression by dehydration were selected for verification of the microarray data using real-time quantitative PCR (RT-qPCR) (Supplementary Table S8). Results shown in Figure 2 clearly demonstrated the reliability of the microarray data.

### Distribution of the dehydration-responsive gene sets identified in W82 and DT2008 roots into functional categories

**Figure 3:**
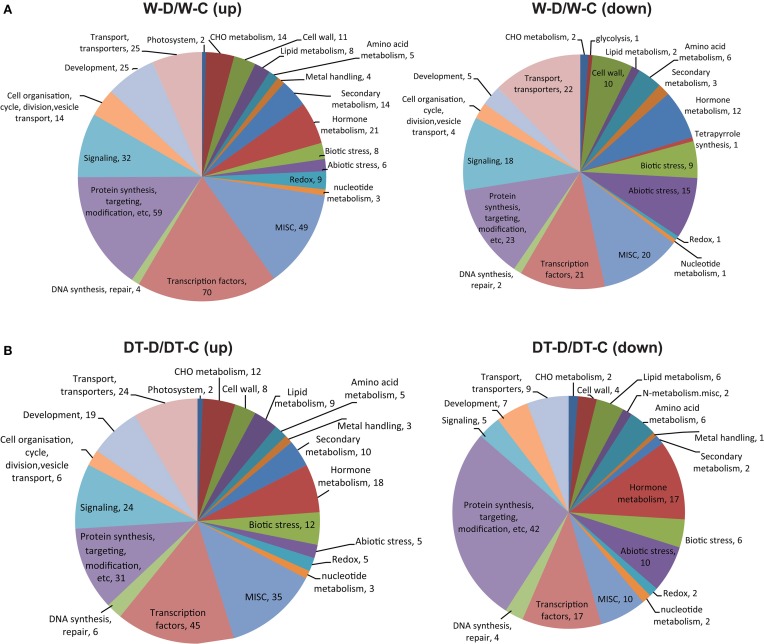
**Distribution of genes differentially expressed in roots of W82 and DT2008 by dehydration treatment into functional categories**. Genes upregulated or downregulated in W82 **(A)** or DT2008 **(B)** roots by at least one dehydration treatment were classified into functional categories using MapMan. W-D/W-C represents W-D2/W-C and/or W-D10/W-C (W82-dehydrated-2 and/or 10 h vs. W82-well-watered control-0 h); DT-D/DT-C represents DT-D2/DT-C and/or DT-D10/DT-C (DT2008-dehydrated-2 and/or 10 h vs. DT2008-well-watered control-0 h).

As a means to understand the molecular mechanisms underlying root responses that soybean plants develop to increase their adaptation to drought, we used MapMan to classify the dehydration-responsive genes detected in W82 and DT2008 into various functional categories. Lists of unique genes with putatively predicted function (Supplementary Tables S6E,F, S7E,F), which were found to be upregulated or downregulated in W82 or DT2008 roots by at least one dehydration treatment, either 2 or 10 h treatment, were assembled and subjected to MapMan analyses for assignment of each gene into functional category (Figure 3). Our data indicated that among the 20 most abundant categories, in both W82 and DT2008 roots, the upregulated genes of the TF category were the most highly enriched genes, whereas the downregulated genes were enriched in “protein synthesis, targeting, modification, etc” category.

### Brief description of the dehydration-responsive gene sets identified in W82 and DT2008 roots

**Figure 4:**
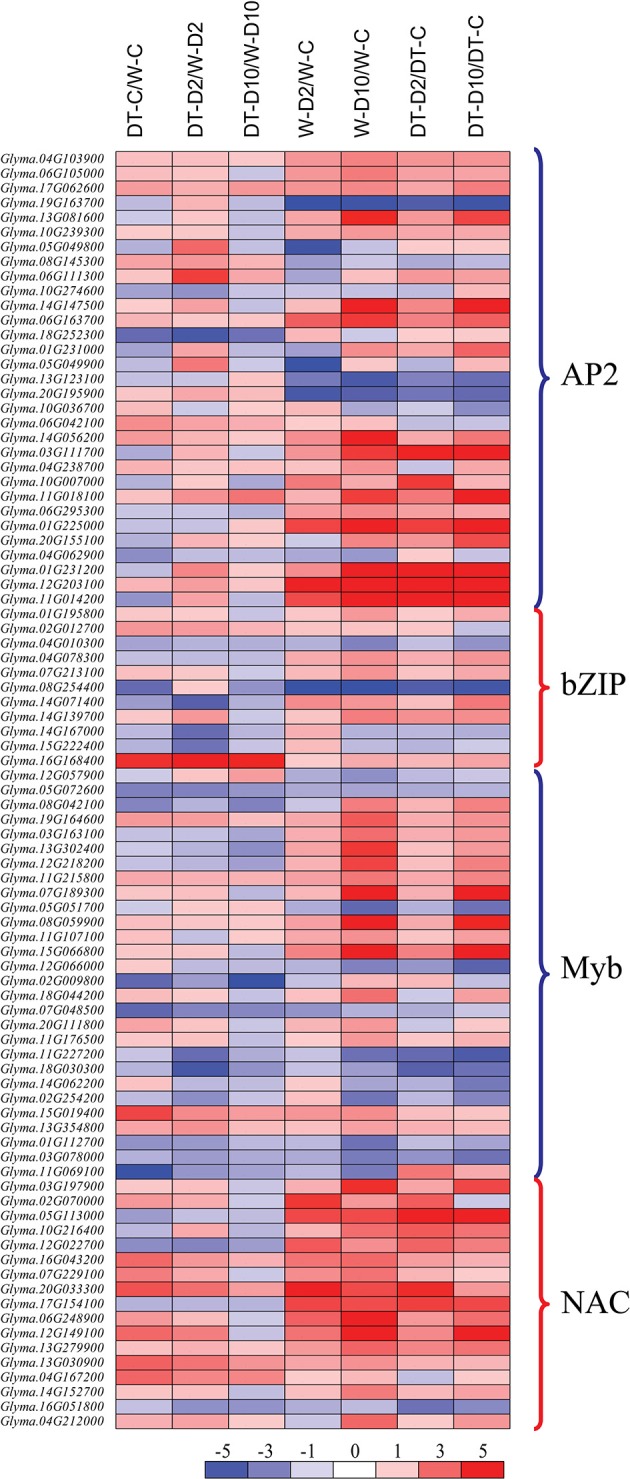
**Heatmap analysis of genes from well-known stress-related transcription factor families showing differential expression in various comparisons under well-watered and/or dehydration conditions**. DT-C/W-C, DT2008-well-watered control-0 h vs. W82-well-watered control-0 h; DT-D2/W-D2, DT2008-dehydrated-2 h vs. W82-dehydrated-2 h; DT-D10/W-D10, DT2008-dehydrated-10 h vs. W82-dehydrated-10 h; W-D2/W-C, W82-dehydrated-2 h vs. W82-well-watered control-0 h; W-D10/W-C, W82-dehydrated-10 h vs. W82-well-watered control-0 h; DT-D2/DT-C, DT2008-dehydrated-2 h vs. DT2008-well-watered control-0 h; DT-D10/DT-C, DT2008-dehydrated-10 h vs. DT2008-well-watered control-0 h.

A closer look at the sets of the DEG sets identified in W82 and DT2008 roots under dehydration revealed a number of common phenomena between their up- and downregulated gene sets, respectively (comparisons W-D/W-C and DT-D/DT-C) (Supplementary Tables S6E,F, S7E,F). Many genes belonging to different TF families, such as the AP2_EREBP-, bZIP-, MYB- and NAC-type TF families, exhibited transcriptional changes by dehydration in both W82 and DT2008 roots, of which more dehydration-inducible genes were found than dehydration-repressible genes (W-D2/W-C, W-D10/W-C, DT-D2/DT-C, and DT-D10/DT-C in Figure 4; Supplementary Figure S2; Supplementary Table S9). For instance, there were 13 and 5 upregulated *GmNAC* genes, in dehydrated W82 and DT2008 roots, respectively, while there were only 0 and 1 downregulated *GmNAC* genes detected in the respective root samples (Supplementary Table S9). Another example is that among the AP2_EREBP-type members, 13 and 11 dehydration-induced genes were found in W82 and DT2008 roots, respectively, in comparison with 3 and 4 dehydration-repressed genes in the respective dehydrated roots (Supplementary Table S9). Under our stringently set criteria of the fold change and *q*-values, the majority of the TF genes of these representative TF families were observed to be induced in either W82 or DT2008 roots by the prolonged 10 h rather than the short 2 h dehydration treatment (Figure 4; Supplementary Table S9).

Apart from the regulatory TFs, a number of DEGs encoding other types of regulatory proteins, such as kinases and hormone signaling-related proteins, were found in the signaling and protein modification categories. Some of them were predicted to be SnRK (sucrose non-fermenting-related), RLK (receptor-like), and MAP (mitogen-activated protein) kinases and PP2C (protein phosphatase 2C) proteins based on sequence homology with their *Arabidopsis* counterparts (Supplementary Figure S2; Supplementary Tables S6E,F, S7E,F). These proteins have been shown to be involved in regulation of plant responses to various stresses, including drought (Umezawa, 2011; Osakabe et al., 2013). Among many dehydration-inducible genes coding for non-regulatory proteins are those encoding proteins of transporters, osmoprotectant biosynthesis-related proteins, and detoxification enzymes. Some are deserved to be mentioned, such as ABC (ATP-binding cassette) transporters, the ABA-importing transporter 1 (AIT1)-like proteins that might have ABA importer activity (Kanno et al., 2012), aquaporins, galactinol synthases, and polyamine oxidases (Supplementary Tables S6E, S7E). An appropriate change of their levels during stress may lead to a better adaptation of the plants (Osakabe et al., 2013; Himuro et al., 2014; Minocha et al., 2014; Rangan et al., 2014; Srivastava et al., 2014).

### Differential expression between W82 and DT2008 roots under normal and dehydration conditions—the upregulated gene sets

**Figure 5:**
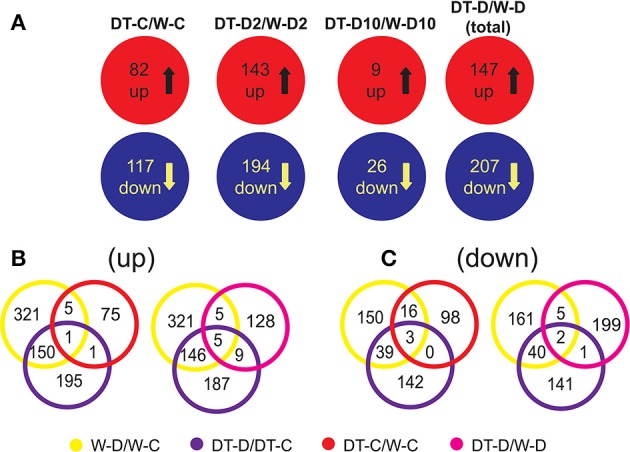
**Comparison of root transcriptomes of W82 or DT2008 under well-watered or dehydration conditions**. **(A)** Differentially express gene sets between W82 or DT2008 roots under well-watered or dehydration conditions. **(B,C)** Identification of dehydration-responsive genes in differentially expressed gene sets that are derived from comparison of root transcriptomes of W82 or DT2008 under well-watered or dehydration conditions. DT-C/W-C, DT2008-well-watered control-0 h vs. W82-well-watered control-0 h; DT-D2/W-D2, DT2008-dehydrated-2 h vs. W82-dehydrated-2 h; DT-D10/W-D10, DT2008-dehydrated-10 h vs. W82-dehydrated-10 h; W-D/W-C represents W-D2/W-C and/or W-D10/W-C (W82-dehydrated-2 h and/or 10 h vs. W82-well-watered control-0 h); DT-D/DT-C represents DT-D2/DT-C and/or DT-D10/DT-C (DT2008-dehydrated-2 h and/or 10 h vs. DT2008-well-watered control-0 h); DT-D/W-D represents DT-D2/W-D2 (DT2008-dehydrated-2 h vs. W82-dehydrated-2 h) and/or DT-D10/W-D10 (DT2008-dehydrated-10 h vs. W82-dehydrated-10 h).

To study the correlation between the differential gene expression in roots of W82 and DT2008 and their differential drought tolerance, we first compared their root transcriptomes under both normal and dehydration conditions. With regard to the upregulated gene sets derived from DT2008 vs. W82 comparison, we found that under well-watered conditions, 82 genes were upregulated in DT-C/W-C comparison, whereas under dehydration, a total of 147 genes were upregulated in DT-D/W-D comparison, with more induced genes being identified during earlier stress (Figure 5A). Namely, 143 upregulated genes were found in DT-D2/W-D2, while only nine upregulated genes in DT-D10/W-D10 (Figure 5A; Supplementary Tables S10A–D). A number of upregulated genes identified in DT-C/W-C and DT-D/W-D comparisons possess putative regulatory functions, as they encode transcription factor, kinase and hormone-related proteins (Supplementary Figure S3).

Next, to identify genes that might contribute to higher drought-tolerant level of DT2008, we first searched for genes that are more highly expressed in drought-tolerant DT2008 than drought-sensitive W82 under normal conditions and are dehydration-inducible in W82 and/or DT2008 roots. We, therefore, subjected the upregulated gene sets obtained from the following comparisons DT-C/W-C, W-D/W-C, and DT-D/DT-C to a Venn analysis (DT-C/W-C vs. W-D/W-C, DT-C/W-C vs. DT-D/DT-C, DT-C/W-C vs. W-D/W-C vs. DT-D/DT-C) (Supplementary Tables S11A–C). As shown in Figure 5B, out of 82 genes displaying higher expression in DT2008 roots than in W82 roots (DT-C/W-C comparison, Supplementary Table S10A), six and two genes were found to be inducible by dehydration in W82 and DT2008 roots, respectively, with one gene, *Glyma.04G083000*, was upregulated in both dehydrated W82 and DT2008 roots (Supplementary Tables S11A–C). Furthermore, genes showing higher expression in DT2008 roots than in W82 roots under dehydration conditions, and being dehydration-inducible in W82 and/or DT2008 roots, might also have impact on improved drought-tolerant level of DT2008 vs. W82. Thus, the upregulated gene sets of DT-D/W-D, W-D/W-C, and DT-D/DT-C comparisons were also evaluated by a Venn analysis. Among 147 genes with more abundant transcripts in DT2008 roots than in W82 roots (DT-D/W-D, Supplementary Table S10D), 10 and 14 genes were detected to be upregulated in dehydrated W82 and DT2008 roots, respectively, of which five genes were upregulated in roots of both cultivars by dehydration (Figure 5B, Supplementary Tables S11D–F).

### Differential expression between W82 and DT2008 roots under normal and dehydration conditions—the downregulated gene sets

As for the downregulated gene sets obtained from comparative analysis of W82 and DT2008 root transcriptomes, we detected 117 and 207 downregulated genes in DT-C/W-C (normal conditions) and DT-D/W-D (dehydration conditions) comparisons, respectively. We also observed that more genes (194 vs. 26 genes) were downregulated in DT-D/W-D comparison by the short-term 2 h (DT-D2/W-D2) rather than the prolonged 10 h (DT-D10/W-D10) dehydration treatment (Figure 5A; Supplementary Tables S10E–H).

In a similar manner, the downregulated gene sets obtained from the comparative analysis of root transcriptomes of W82 and DT2008 under non-stressed and stressed conditions (DT-C/W-C and DT-D/W-D) were also analyzed to identify dehydration-repressible genes exhibiting lower expression in drought-tolerant DT2008 as these genes would also be responsible for better performance of DT2008 relative to W82 under drought. Thus, downregulated gene sets of DT-C/W-C, DT-D/W-D, W-D/W-C, and DT-D/DT-C comparisons were evaluated by a Venn analysis as well (Supplementary Table S12). Venn diagrams shown in Figure 5C indicated that a total of 19 genes had lower expression in DT2008 roots than in W82 roots under well-watered conditions. All these 19 genes were repressed by dehydration in W82 roots, of which three genes were also downregulated in DT2008 roots (Supplementary Tables S12A–C). As for genes showing lower expression levels in DT2008 roots than W82 roots under stress conditions, we found a total of eight genes of which five and one genes were repressed by dehydration in W82 or DT2008 roots only, while two genes were dehydration-repressed in roots of both cultivars (Figure 5C, Supplementary Tables S12D–F).

## Discussion

Large-scale transcriptome analysis is one of the most comprehensive approaches used to identify gene repertoire whose members are responsible to certain stressors (Mochida and Shinozaki, 2011). The completion of soybean genomic sequence has enabled us to carry out high-throughput transcriptomic studies in this important legume crop under various stress conditions in different organs (Schmutz et al., 2010; Le et al., 2012b; Ferreira Neto et al., 2013; Wan et al., 2015). Genes identified through the large-scale expression profiling studies, not only in soybean but also in other crops, have significantly accumulated in the past decade, providing a valuable resource for further functional genomics and comparative analyses (Ma et al., 2012).

DT2008 is an elite soybean cultivar cultivated in many regions in Vietnam, owing to its strong tolerance to drought and dehydration in comparison with many other cultivars (Vinh et al., 2010; Ha et al., 2013; Sulieman et al., 2015). In a previous study, we compared the drought tolerability of DT2008 and the W82 model cultivar, and found that the higher drought-tolerant degree of DT2008 relative to W82 might be attributed, at least, to its better root development in comparison with W82 (Ha et al., 2013). To explain this phenomenon at molecular level, in the current study we carried out a microarray analysis of root transcriptomes of both DT2008 and W82 under normal, as well as mild (2 h-treated) and severe (10 h-treated) dehydration stress conditions using the 66K soybean GeneChip (Figures 1A,B, Supplementary Table S1). This custom 66K Affymetrix GeneChip has been shown to be a reliable tool for large-scale gene expression analysis in different organs under different types of stress, such as leaves (Le et al., 2012b) and roots (this work) under drought/dehydration stress, and in the same organs under biotic stress (Valdes-Lopez et al., 2011; Wan et al., 2015).

With the release of the newest annotation version Glyma v2.0 (http://phytozome.jgi.doe.gov/pz/portal.html), we were able to examine the expression of 38172 genes with high confidence through our transcriptome analysis (Supplementary Table S2). In general, we found more DEGs in roots of drought-sensitive W82 than in that of drought-tolerant DT2008 under dehydration in both upregulated and downregulated categories. Specifically, 2.15% (822/38172 genes) of the 38172 examined genes, which were annotated with high confidence, showed altered expression by dehydration in W82 roots, whereas 1.66% (632/38172 genes) of the analyzed genes exhibited differential expression in DT2008 roots under the same treatment conditions (Supplementary Figure S1A). On the other hand, in another independent study using DeepSuperSAGE (26 bp tags) for comparative root transcriptome analysis of 15-day-old drought-tolerant Embrapa 48 and drought-sensitive BR 16 seedlings at early stage of dehydration stress (between 0 and 150 min with 25 min interval), the authors in total found more differentially expressed soybean unitags in drought-tolerant Embrapa 48 roots than in drought-sensitive BR 16 roots in both upregulated and downregulated categories (Ferreira Neto et al., 2013). These findings suggest that different varieties might transcriptionally respond to dehydration/drought in different ways to activate root-related mechanisms for higher tolerability when compared with a specific drought-sensitive genotype. Alternatively, the different growth conditions might be a reason for the different observations of the two studies, as we grew the soybean plants in soil, whereas Ferreira Neto and colleagues hydroponically cultivated their soybean plants in nutrient solution (Ferreira Neto et al., 2013). It is worthy to notice that we also detected more upregulated genes in drought-tolerant DT2008 roots than drought-sensitive W82 roots by early 2 h dehydration treatment (131 vs. 105), although a reverse tendency was observed in case of downregulated genes (34 vs. 47) (Figure 1C, Supplementary Figure S1A). These results together suggest that induction of more dehydration/drought-responsive genes in roots of drought-tolerant cultivars, as compared with that in drought-sensitive cultivar, at early stage of stress exposure might contribute to its higher drought tolerability (Vinh et al., 2010; Ferreira Neto et al., 2013; Ha et al., 2013).

In addition, we recorded more DEGs in roots of both DT2008 (DT-D10/DT-C vs. DT-D2/DT-C) and W82 (W-D10/W-C vs. W-D2/W-C) by 10 h than 2 h dehydration treatment (Figure 1C, Supplementary Figure S1A). These data indicated that the long-term dehydration stress triggered change in expression of more genes in soybean roots than the short-term one, independently of the genotype. Furthermore, the MAPMAN analysis showed that TF encoding genes were the most highly enriched upregulated genes, whereas those classified to “protein synthesis, targeting, modification, etc” category were the most highly enriched downregulated genes in both W82 and DT2008 roots under dehydration (Figure 3). This finding suggested that genes belonging to these categories were those whose expression in roots is the most responsive to dehydration to aid the plants in adapting to the stress. Interestingly, a previous microarray analysis using the same GeneChip found a reverse trend in V6 and R2 leaves of the W82 cultivar. The authors reported that in these W82 leaf tissues, TF encoding genes were enriched among the downregulated genes; while, for the upregulated gene sets, “protein synthesis, targeting, modification, etc” was the most significantly enriched category (Le et al., 2012b).

With respect to the TF encoding genes, many members of the major TF families, such as AP2_EREBP, bZIP, MYB, and NAC, showed differential expression by dehydration in both W82 and DT2008 roots (Figure 4). Moreover, the heatmap analysis also indicated that the majority of the dehydration-inducible TF genes, such as *NAC* genes, exhibited higher expression level in DT2008 roots than W82 roots, especially under well-watered and early dehydration treatment (Figure 4). Increasing evidence has shown that members of these TF families play important roles in plant responses to water deficit by controlling transcription of downstream genes through their specific binding to the so-called *cis*-acting elements located in the promoters of target genes (Yamaguchi-Shinozaki and Shinozaki, 2006; Hadiarto and Tran, 2011; Jogaiah et al., 2013). A number of published reports have shown positive correlation between *NAC* gene expression levels, specifically in roots or leaves or whole plants, and drought tolerability of various crops, including soybean (Nakashima et al., 2007; Zheng et al., 2009; Xue et al., 2011; Thao et al., 2013; Thu et al., 2014; Zhu et al., 2014; Nguyen et al., 2015; Yang et al., 2015), further supporting that NAC TFs, at least in part, might contribute to the higher drought tolerance of DT2008 vs. W82. Molecular tailoring of the TF encoding genes has provided a promising approach for improvement of tolerance of a number of crops to various types of environmental stresses, including drought (Yang et al., 2010; Thao and Tran, 2012).

Apart from the TF genes, many other dehydration-inducible genes also displayed higher expression levels in DT2008 roots than W82 roots under normal or dehydration conditions (Supplementary Table S11), which might contribute to differential drought tolerance of DT2008 and W82. Results summarized in Figure 5 indicated that the short-term dehydration-induced expression changes might be more highly required for enhanced drought tolerance of DT2008 vs. W82 than the prolonged dehydration-induced ones, as significantly higher number of DEGs were identified in DT-D2/WT-D2 comparison than DT-D10/WT-D10 comparison. With regard to dehydration-upregulated genes with higher expression levels in DT2008 roots vs. W82 roots under well-watered conditions (Supplementary Tables S11A–C), *Glyma.14G216500* encodes an ortholog STH2 (salt tolerance homolog2) (Table 1), a B-box TF that can act as a positive regulator of photomorphogenesis and anthocyanin biosynthesis (Datta et al., 2007). This gene might play a role in enhanced tolerance of DT2008 as anthocyanins are known to protect plants against various environmental stresses, including drought (Pourcel et al., 2007). Modulation of the signaling molecule phospholipids, which involved in regulation of plant response to environmental stimuli, through *Glyma.04G083000* (induced by dehydration in both DT2008 and W82 background, Table 1) and *Glyma.20G189100* that encode putative proteins with function in phosphoinositide signaling, might also be responsible for increased tolerance of DT2008 to drought (Liu et al., 2013). As for the dehydration-inducible genes showing higher transcription levels in DT2008 roots vs. W82 roots under dehydration (Supplementary Tables S11D–F), *Glyma.19G227800, Glyma.16G003500*, and *Glyma.13G095200* encoding osmoprotectant biosynthesis-, detoxification- or cell wall-related proteins (Table 1), such as the orthologs of *Arabidopsis* AtGOLS2 (*Arabidopsis thaliana* galactinol synthase 2), glyoxalase I family protein and xyloglucan endotransglycosylase, may play important roles in better adaptation of DT2008 to drought relative to W82 as supported by previous studies (Xu et al., 1995; Taji et al., 2002; Kaur et al., 2014).

**Table 1:** **List of several candidate genes that might contribute to higher drought tolerance of DT2008 vs. W82**.

**Glyma 2 ID**	***Arabidopsis* ortholog**	**Description**	**W-D/W-C**	**DT-D/DT-C**	**DT-C/W-C**	**DT-D2/W-D2**	**DT-D10/W-D10**
			**Responsiveness**	**Responsiveness**	**Fold change (*q* < 0.05)**	**Fold change (*q* < 0.05)**	**Fold change (q < 0.05)**
*Glyma.04G083000*	*AT3G22810*	Putative phosphoinositide binding protein	Induced	Induced	2.61	Unchanged	Unchanged
*Glyma.20G189100*	*AT3G10550*	Putative inositol or phosphatidylinositol phosphatase	Induced	None	2.03	Unchanged	Unchanged
*Glyma.14G216500*	*AT1G75540*	Similar to STH2 (salt tolerance homolog2)	Induced	None	4.40	Unchanged	Unchanged
*Glyma.13G095200*	*AT4G25810*	Similar to XTR6 (xyloglucan endotransglycosylase 6)	None	Induced	Unchanged	2.16	Unchanged
*Glyma.16G003500*	*AT1G80160*	Similar to GLYI7 (glyoxylase I 7)	Induced	None	Unchanged	2.91	Unchanged
*Glyma.19G227800*	*AT1G56600*	Similar to AtGOLS2 (*A.thaliana* galactinol synthase 2)	Induced	Induced	Unchanged	3.70	Unchanged
*Glyma.02G069400*	*AT4G18710*	Similar to BIN2 (brassinosteroid-insensitive 2)	Repressed	Repressed	−3.56	Unchanged	Unchanged
*Glyma.06G050900*	*AT5G36250*	Similar to protein phosphatase 2C 74	Repressed	Repressed	−3.23	−2.30	Unchanged
*Glyma.08G254400*	*AT3G30530*	Similar to bZIP42	Repressed	Repressed	Unchanged	Unchanged	−2.05
*Glyma.12G202400*	*AT4G03415*	Similar to protein phosphatase 2C 52	Repressed	None	−2.11	Unchanged	Unchanged

W-D/W-C represents W-D2/W-C and/or W-D10/W-C (W82-dehydrated-2 h and/or 10 h vs. W82-well-watered control-0 h); DT-D/DT-C represents DT-D2/DT-C and/or DT-D10/DT-C (DT2008-dehydrated-2 h and/or 10 h vs. DT2008-well-watered control-0 h); DT-D2/W-D2, DT2008-dehydrated-2 h vs. W82-dehydrated-2 h; DT-D10/W-D10, DT2008-dehydrated-10 h vs. W82-dehydrated-10 h.

Additionally, dehydration-repressible genes with lower expression level in drought-tolerant DT2008 roots than drought-sensitive W82 might also contribute to the better performance of DT2008 vs. W82 under drought. Among the dehydration-repressible genes that had lower expression levels in DT2008 than W82 under well-watered conditions (Supplementary Tables S12A–C), *Glyma.02G069400* codes for an ortholog of *Arabidopsis* BIN2 (brassinosteroid-insensitive 2) (Table 1). It was reported that the rice ortholog of BIN2, the OsGSK1 (glycogen synthase 3-like protein kinase), acts as a negative regulator of plant responses to multiple stresses, including drought (Koh et al., 2007). Repression of *Glyma.02G069400* might therefore contribute to increased drought tolerance of DT2008. Another example is *Glyma.12G202400* encoding a protein with high homology to an *Arabidopsis* phosphatase 2C protein (AT4G03415) (Table 1) that might be involved in drought response perhaps through its interaction with CPK16 of Ca^2+^-dependent protein kinase/sucrose non-fermenting related kinase (CPK/SnRK) superfamily (Curran et al., 2011). CPK16 has been known to be implicated in regulation of root gravitropism that is a trait important for plant response to water stress (Kirkham, 2008; Huang et al., 2013). With regard to the dehydration-repressible genes that displayed lower expression levels in DT2008 roots than W82 roots under dehydration (Figure 5C; Supplementary Tables S12D–F), *Glyma.06G050900* and *Glyma.08G254400* encoding phosphatase 2C and bZIP orthologs of *Arabidopsis*, respectively, caught our attention (Table 1). *Glyma.06G050900* exhibited lower expression in DT2008 roots than W82 roots under both normal and dehydration conditions (Table 1). It is well established that many members of the phosphatase 2C family are involved in stress signaling (Schweighofer et al., 2004). Several phosphatase 2C proteins have been found to act as negative regulators of ABA signaling (Umezawa et al., 2010). As for *Glyma.08G254400*, although there is no specific information either for this gene or its highest *Arabidopsis* homolog (AT3G30530/AtbZIP42), there have been published reports that several bZIP TFs act as negative regulators of drought tolerance. For instance, overexpression of *OsbZIP52* in rice significantly enhanced sensitivity of transgenic plants to cold and drought stresses (Liu et al., 2012). Thus, downregulation of such a gene would allow plants to adapt better to adverse environmental conditions.

In summary, our comparative analysis of root transcriptomes of DT2008 and W82 under both well-watered and dehydration conditions have allowed us to identify genetic components that might contribute to the improved drought tolerance of DT2008. Our study also provides a useful genetic resource for scientists with interests in basic and/or applied research to carry out further in-depth gene characterization and functional analyses. This in turn will contribute to deeper understanding of mechanisms regulating drought responses and adaptation in soybean, which ultimately leads to development of soybean cultivars with improved tolerance to drought.

## Materials and methods

### Plant growth and dehydration treatment

W82 and DT2008 soybean plants were separately grown in pots containing vermiculite (6 plants per 6-liter pot) under well-watered conditions in a controlled greenhouse (continuous 30°C temperature, photoperiod of 12/12 h, 150 μmol m^−2^ s^−1^ photon flux density). For the collection of well-watered and dehydrated root tissues, 14-d-old soybean plants with two trifoliate leaves (V2 stage) were carefully removed from pots, then gently washed to remove soil from the roots. Subsequently, the W82 and DT2008 plants were dried on a filter paper for different time periods under the condition of 44% relative humidity, 23°C room temperature and 10 μmol m^−2^ s^−1^ photon flux light intensity. The severity of the stress level was measured by determination of RWC of the aerial parts of dehydrated plants. After the dehydration treatment, plants dehydrated for 0, 2, and 10 h were collected and the roots were separated from the shoots. Root samples were quickly frozen in liquid nitrogen and stored at −80°C until RNA purification. Accordingly, the following root samples were collected in three biological replicates from W82 and DT2008 plants for microarray analysis: W82 well-watered control 0 h (W-C), W82 dehydrated 2 h (W-D2), W82 dehydrated 10 h (W-D10), DT2008 well-watered control 0 h (DT-C), DT2008 dehydrated 2 h (DT-D2); DT2008 dehydrated 10 h (DT-D10).

### Microarray analysis of the root samples using 61K affymetrix microarray

RNAs were extracted from root samples using the Trizol reagent (Invitrogen, Carlsbad, CA, USA) as recommended by the manufacturer's protocol. Purified total RNA was subsequently subjected to a DNase I treatment prior to the quality assessment by an Agilent 2100 Bioanalyzer (Le et al., 2011a). For microarray analysis, cDNA synthesis, cRNA amplification, and synthesis of sense strand cDNAs were carried out using the Ambion WT expression kit. cDNA labeling was carried out using Affymetrix GeneChip WT Terminal Labeling Kit according to the supplier's instructions. Hybridization and scanning of hybridized arrays (G2505B microarray scanner, Agilent Technologies) were performed as described previously (Nishiyama et al., 2012). Three biological replicates collected from each treatment were subjected to the microarray experiment. Microarray data were analyzed using Affymetrix Expression Console with library supplied from Affymetrix and GeneSpring (Ver. 11) as essentially described (Le et al., 2012b). Statistical significance of each gene in each treatment (*p*-value) was estimated by a Student's *t*-test, while its certainty level (the corrected *p*-values, i.e., *q*-values) was assessed using Benjamini and Hochberg False Discovery Rate. Genes with expression change ≥2-fold (*q* < 0.05) were regarded to be differentially expressed. The obtained microarray data have been deposited in the Gene Expression Omnibus (GEO) database (http://www.ncbi.nlm.nih.gov/geo/browse/?view=series) (accession number GSE65553)^1^.

### MapMan analysis of the root transcriptomes

MapMan (http://mapman.gabipd.org) was used to annotate and analyze the microarray data as according to previously published methods (Thimm et al., 2004; Le et al., 2012b; Nishiyama et al., 2012; Ha et al., 2014). The lists containing DEGs obtained from corresponding comparisons were supplied to MapMan for classification of DEGs into functional groups.

### Validation of microarray data by RT-qPCR

Several genes were randomly selected for verification of the microarray data using RT-qPCR.

The specific primer pairs used in RT-qPCR were listed in Supplementary Table S8. The *Fbox* gene was used as a reference gene in the RT-qPCR analysis of RNA samples from three biological replicates (Le et al., 2012a). Preparation of cDNAs from DNase I-treated RNA samples for RT-qPCR was performed as previously described (Le et al., 2011b).

## Author contributions

L-SPT conceived research and wrote the manuscript. CVH, YW, UTT, DTL, MT, and KHN performed the experiments and analyzed the data. MS and DVN contributed research materials.

### Conflict of interest statement

The authors declare that the research was conducted in the absence of any commercial or financial relationships that could be construed as a potential conflict of interest.

## Footnotes

^1^Accession number to microarray data deposited at Gene Expression Omnibus database: GSE65553.
