# Supplementary material for: Comparative analysis of root transcriptomes from two contrasting drought-responsive Williams 82 and DT2008 soybean cultivars under normal and dehydration conditions
Source: Front Plant Sci. 2015 Aug 7;6:551. doi: 10.3389/fpls.2015.00551 (PMC4528160; doi:10.3389/fpls.2015.00551)
Supplement: Supplementary file 13 [file Presentation1.PDF]

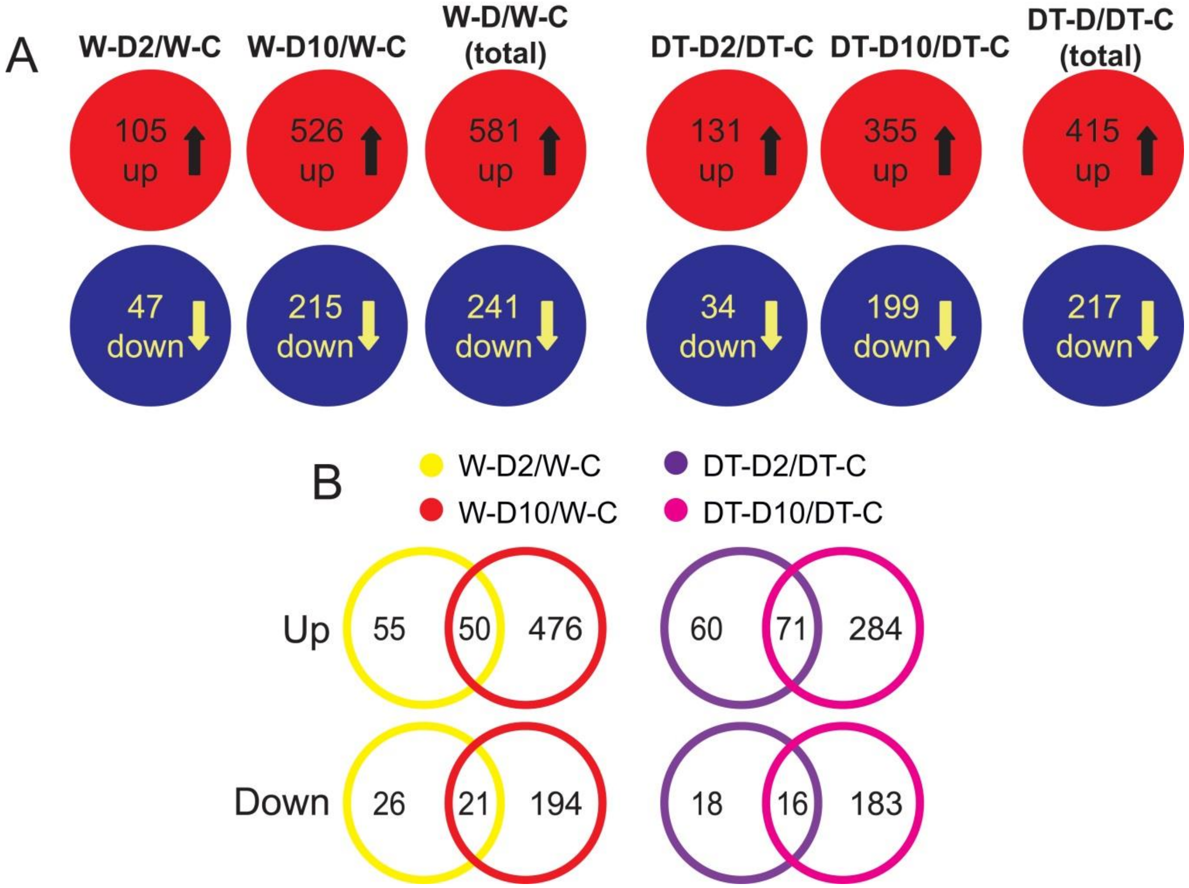

Supplementary Figure S1. **Summary of the results of the microarray analysis**. (A) Upregulated and downregulated genes identified in each comparison from 38172 genes annotated by Glyma v2.0 with high confidence. Data were obtained from the results of three independent microarray experiments of three biological repeats. (B) Effect of stress intensity on gene expression in roots of W82 and DT2008 as indicated by Venn analysis of differentially expressed gene sets identified in (A).

W-D2/W-C, W82–dehydrated-2h vs. W82–well-watered control-0h; W-D10/W-C, W82–dehydrated-10h vs. W82–well-watered control-0h; W-D/W-C represents W-D2/W-C and/or W-D10/WC (W82–dehydrated-2h and/or 10h vs. W82–well-watered control-0h); DT-D2/DT-C, DT2008–dehydrated-2h vs. DT2008–well-watered control-0h; DT-D10/DT-C, DT2008–dehydrated-10h vs. DT2008–well-watered control-0h; DT-D/DT-C represents DT-D2/DT-C and/or DT-D10/DT-C (DT2008–dehydrated-2h and/or 10h vs. DT2008–well-watered control-0h.

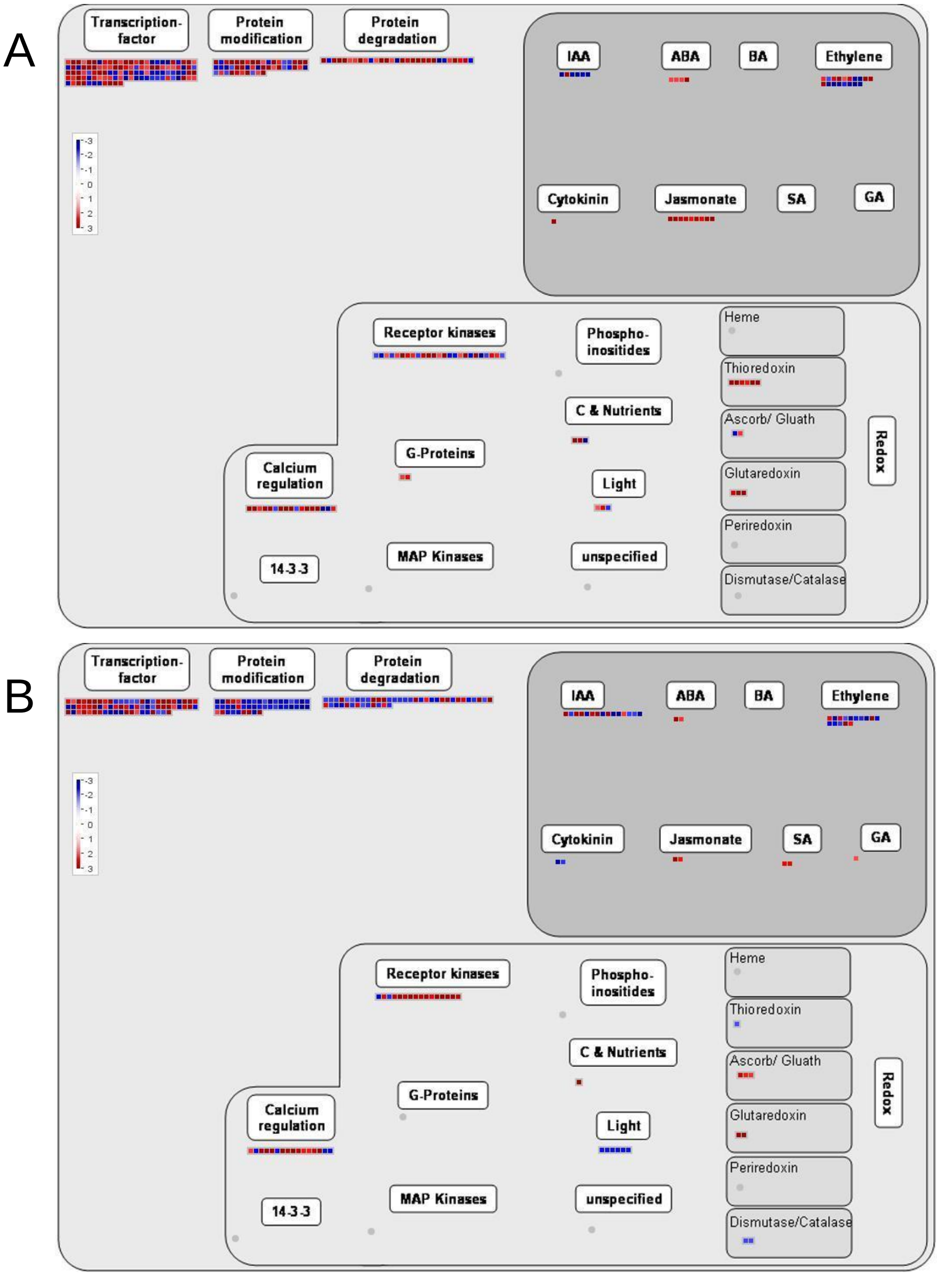

Supplementary Figure S2. Diagrammatic representation of regulation-related DEGs obtained from microarray analysis of DT2008 and W82 roots under normal and dehydration conditions. (A) DEGs from W-D/W-C comparison were used in MAPMAN analysis. (B) DEGs from DT-D/DT-C comparison were used in MAPMAN analysis. Blue and red colors indicate downregulation and upregulation, respectively. Fold changes in gene expression were indicated by colored bars in each panel.

W-D/W-C represents W-D2/W-C and/or W-D10/WC (W82–dehydrated-2h and/or 10h vs. W82–well-watered control-0h); DT-D/DT-C represents DT-D2/DT-C and/or DT-D10/DT-C (DT2008–dehydrated-2h and/or 10h vs. DT2008–well-watered control-0h).

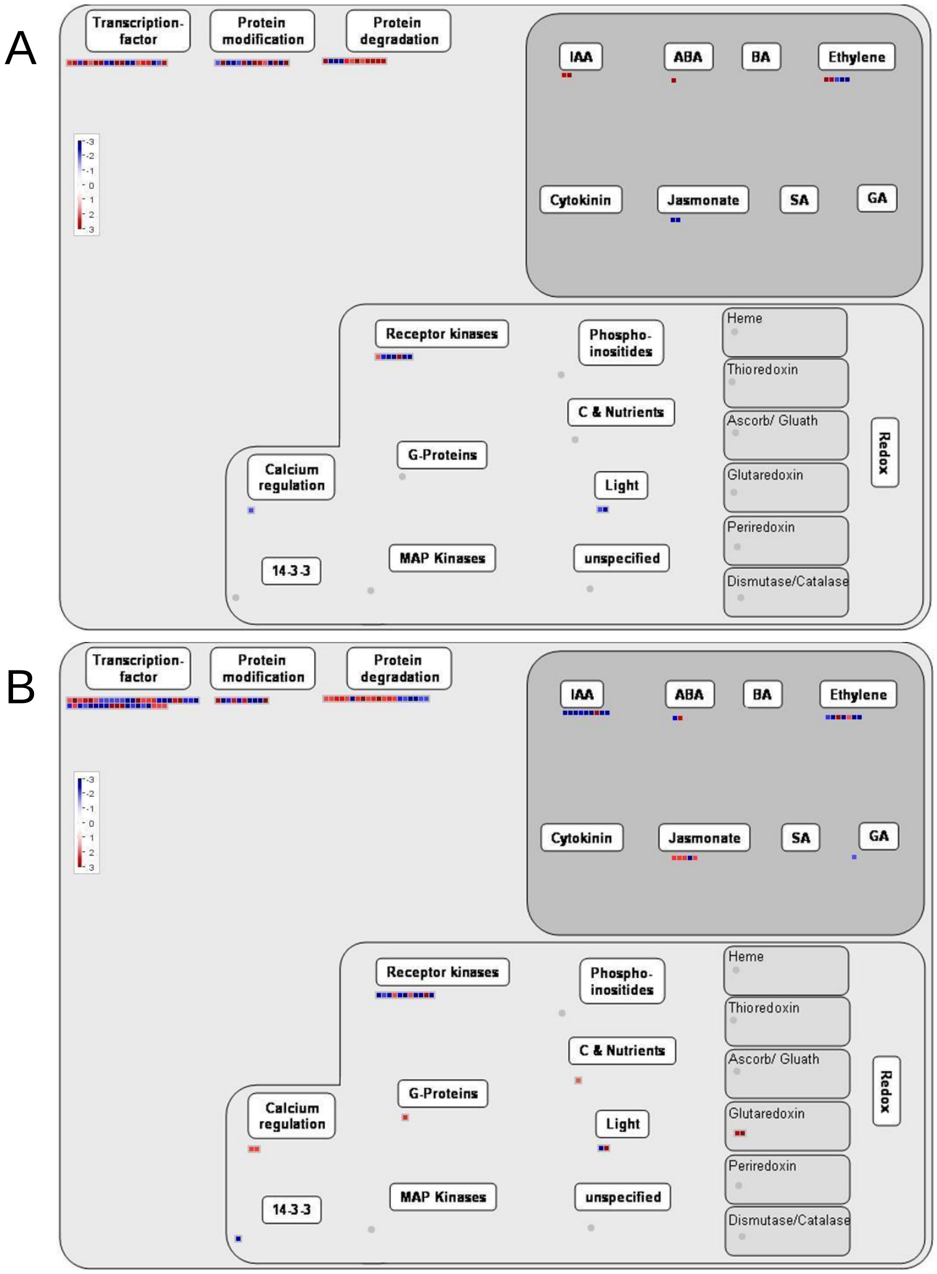

Supplementary Figure S3. Diagrammatic representation of regulation-related DEGs obtained from comparative analysis of DT2008 and W82 root transcriptomes. (A) DEGs from DT-C/W-C comparison were used in MAPMAN analysis. (B) DEGs from DT-D/W-D comparison were used in MAPMAN analysis. Blue and red colors indicate downregulation and upregulation, respectively. Fold changes in gene expression were indicated by colored bars in each panel. DT-C/W-C, DT2008–well-watered control-0h vs. W82–well-watered control-0h; DT-D/W-D represents DT-D2/W-D2 (DT2008–dehydrated-2h vs. W82–dehydrated-2h) and/or DT-D10/W-D10 (DT2008–dehydrated-10h vs. W82–dehydrated-10h).
